# Atomistic Interpretation of the Oxygen K-Edge X-ray Absorption Spectra of Layered Li-Ion Battery Cathode Materials

**DOI:** 10.1021/acs.chemmater.4c01870

**Published:** 2024-11-12

**Authors:** Namrata Ramesh, Hrishit Banerjee, Jack E. N. Swallow, Erik Björklund, Ava Dean, Pravin Didwal, Michael Fraser, Conor M. E. Phelan, Lijin An, Jasper Singh, Jarrod Lewis, Weixin Song, Robert A. House, Andrew J. Morris, Robert S. Weatherup, Rebecca J. Nicholls

**Affiliations:** † Department of Materials, Oxford University, Oxford OX1 3PH, U.K.; ‡ Yusuf Hamied Department of Chemistry, University of Cambridge, Cambridge CB2 1EW, U.K.; § School of Science and Engineering, University of Dundee, Scotland DD1 4HN, U.K.; ∥ The Faraday Institution, Quad One, Harwell Science and Innovation Campus, Didcot OX11 0RA, U.K.; ⊥ Department of Physics, University of York, York YO10 5DD, U.K.; # School of Metallurgy and Materials, University of Birmingham, Birmingham B15 2SE, U.K.

## Abstract

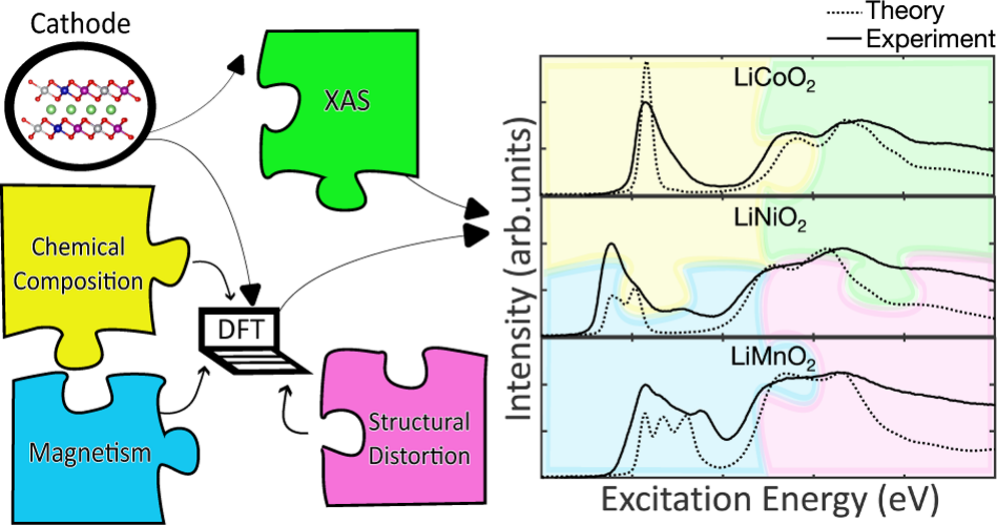

Core loss spectroscopies can provide powerful element-specific insight into the redox processes occurring in Li-ion battery cathodes, but this requires an accurate interpretation of the spectral features. Here, we systematically interpret oxygen K-edge core loss spectra of layered lithium transition-metal (TM) oxides (LiMO_2_, where M = Co, Ni, Mn) from first principles using density-functional theory (DFT). Spectra are simulated using three exchange–correlation functionals, comprising the generalized gradient approximation (GGA) functional PBE, the DFT–PBE + Hubbard *U* method, and the *meta*-GGA functional rSCAN. In general, rSCAN provides a better match to experimentally observed excitation energies of spectral features compared to both PBE and PBE + *U*, especially at energies close to the main edge. Projected density of states of core-hole calculations show that the O orbitals are better described by rSCAN. Hybridization, structural distortions, chemical composition, and magnetism significantly influence the spectra. The O K-edge spectrum of LiNiO_2_ obtained using rSCAN shows a closer match to the experimental X-ray absorption spectroscopy (XAS) when derived from a simulation cell which includes a Jahn–Teller distortion, showing that the DFT-calculated pre-edge feature contains information about not only chemical species but also geometric distortion. Core loss spectra derived from DFT can also differentiate between materials with the same structure and magnetic configuration but comprising different TMs; these differences are comparable to those observed in experimental XAS from the same materials. This foundational work helps establish the extent to which DFT can be used to bridge the interpretation gap between experimental spectroscopic signatures and ab initio methods describing complex battery materials, such as lithium nickel manganese cobalt oxides.

## Introduction

Li-ion batteries (LIBs) are an enabling technology for the decarbonization of transport, given they can be readily integrated with intermittent renewable energy sources.^1−3^ However, widespread adoption of battery electric vehicles requires further improvements in cost and energy density of LIBs, which remain limited by the cathode active material (typically based on LiCoO_2_).^4^ Current efforts to improve performance and capacity involve reducing the Co content of cathode materials by replacing Co with less toxic and less expensive Mn and Ni, resulting in the lithium nickel manganese cobalt oxides (NMC), which also show increased practical capacities, particularly for Ni-rich stoichiometries.^5−8^ The increased complexity of these materials warrants further consideration of how their charge compensation mechanisms vary with composition, including the extent to which different transition metal (TM) centers and oxygen participate during cycling. Improved understanding of oxygen’s role in charge compensation could inform approaches to increase capacity^9,10^ or mitigate degradation of cathode active materials.^5,11^

The dominant experimental techniques for understanding oxygen redox activity in Li-ion battery cathode materials are O K-edge core loss spectroscopies, which involve probing transitions from the O 1s to unoccupied states and include X-ray absorption spectroscopy (XAS),^12,13^ electron energy loss spectroscopy,^12,14^ X-ray Raman spectroscopy, and resonant inelastic X-ray scattering (RIXS).^15,16^ Although RIXS can potentially give detailed insight into the nature of O redox,^17,18^ interpreting RIXS is challenging, even when first-principles calculations are available for comparison.^19,20^ On the other hand, interpretation of O K-edge XAS spectra can be carried out using first-principles methods such as density-functional theory (DFT).^21^

Combining experimental XAS with DFT can provide direct insight into the chemical environments that give rise to different spectral features. Indeed, DFT could be used to atomistically model the core-loss spectra of NMC materials with novel TM ratios or layered Li TM oxides at different states of charge. A step toward systematically modeling these more complex environments is to consider the pristine parent materials of NMC, which include LiCoO_2_, LiNiO_2_, and LiMnO_2_. The O K-edge core-loss spectra of some of these parent materials have been previously modeled using DFT with a variety of exchange–correlation functionals, which take into account varying degrees of electron exchange and correlation (a more comprehensive definition of exchange–correlation functionals is presented in the Computational Details section). The O K-edge core-loss spectrum of LiCoO_2_ has been modeled using the generalized-gradient approximation (GGA) exchange–correlation functional PBE,^22^ the DFT + *U* method with PBE,^14,23,24^ and with hybrid functionals (HSE).^23^ The O K-edge core-loss spectra of LiNiO_2_ and NMC-622 have been modeled using PBE by Genreith-Schriever et al.^25^ and using HSE by Banerjee et al., respectively.^26^ However, there has been no systematic investigation of the effect that different DFT exchange–correlation functionals and the subtle changes in the octahedra have on the theoretical K-edge core-loss spectral shape of the layered Li TM oxides.

In this article, we provide a solid foundation for atomistically interpreting the O K-edge spectra of layered Li TM oxides by varying the exchange–correlation functional used, the chemical identity of the coordinating environment species, and the magnetic configuration of the coordinating environment. Furthermore, we explore the impact of changes in the oxygen environment on spectral characteristics by including for the first time in the reported literature, to our knowledge, both theoretical and experimental O K-edge spectra of monoclinic LiMnO_2_. As it is isostructural with LiCoO_2_ and LiNiO_2_, the theoretical spectra arising from this material serve as a useful comparison for systematically assessing the suitability of DFT for atomistically interpreting the XAS of layered Li TM oxides. The impact of Jahn–Teller (JT) distortion, TM identity, and magnetic ordering on the pre-edge features at the DFT-level is revealed by comparing the theoretical spectra of a range of structure models.

We focus herein on DFT interpretation of the K-edge spectra of the pristine parent materials of NMC to provide a benchmark for future studies of the more complex NMC, which requires larger supercells to capture the stoichiometry and state of charge. Thus, this work highlights the extent to which DFT can be used to atomistically interpret the oxygen K-edge spectra of layered Li TM oxide cathodes, laying the foundation for a methodology that could be used to investigate the role that oxygen plays in the charge compensation mechanism of novel Li-ion battery cathode materials.

## Methods

### Sample Preparation

Samples of three parent materials of NMC, LiCoO_2_, LiNiO_2_, and monoclinic LiMnO_2_ (*m*-LiMnO_2_) were prepared for the O K-edge XAS measurements. LiCoO_2_ and LiNiO_2_ were in composite electrode form, and *m*-LiMnO_2_ was in powder form. All samples were mounted onto the sample plate in an Ar glovebox by using copper adhesive tape. The oxygen and water contents in the glovebox were less than 0.1 ppm. The LiCoO_2_ electrode is a commercially available electrode from MSE Supplies and consists of 95% active material mixed with conductive carbon and poly(vinylidene difluoride) binder, coated onto an aluminum current collector. The LiNiO_2_ electrode was self-standing, consisting of a polycrystalline LiNiO_2_ powder obtained from BASF without deliberate doping/coating. The active material was mixed with conductive carbon and PTFE in an 8:1:1 ratio in a pestle and mortar. The film was then calendered to a thickness of 150 μm. The synthesis of *m*-LiMnO_2_ was based on that developed by Armstrong and Bruce^27^ involving ion exchange of NaMnO_2_. The starting NaMnO_2_ was prepared by heating Na_2_CO_3_ and Mn_2_O_3_ together at 700 °C under an Ar atmosphere for 12 h. As-prepared NaMnO_2_ was then refluxed with an excess of LiBr in *n*-hexanol at 150 °C for 12 h. The resulting *m*-LiMnO_2_ product was filtered, washed with methanol, and dried in a vacuum oven overnight.

### XAS Measurement Details

The XAS data was collected in fluorescence yield (FY) mode using an Al coated Si photodiode at endstation 2 of the B07-B beamline at Diamond with the exit slits set to 50 μm in the dispersive direction. All samples were measured with the incident beam normal to the sample plate, yielding a beam footprint of 150 × 100 μm, and the photodiode directed at the sample with its surface normal at ∼45° to the incident beam direction. The information depth corresponds to an X-ray attenuation length of ∼200 nm at the K-edge energy, providing a reasonably bulk-sensitive measure for comparison with theoretical models of the bulk crystalline structures. The energy resolution ∼0.1 eV.^28^ Energy calibration was performed by comparing the O K-edge XAS of NiO (Alfa Aesar, Puratronic, 99.998%) measurements with that measured by Davoli et al.^29^ The data was normalized by dividing by the mirror current to take into account variations of the beam intensity with energy. A linear background was then subtracted using the region before the first peak, and intensity normalization was performed using the height of the pre-edge peak. The LiNiO_2_ FY data has been previously reported in An et al.^12^.

### Computational Details

Density functional theory makes use of the Hohenberg–Kohn theorem, which proves that the ground state energy is a functional of the electron density.^30^ The theorem may be used in practice via the Kohn–Sham equations, which simplify the problem by representing the total energy functional as a sum of energy contributions, which include contributions from the potential energy, the kinetic energy of noninteracting electrons in an effective potential (mean-field approximation), the Hartree energy (from the interactions of electrons described classically), and a fourth energy term known as the exchange–correlation energy functional *E*_xc_.^31^ The exchange–correlation functional can be approximated as being dependent on the local density, implemented via the local density approximation, or additionally being dependent on the gradient of this density, implemented via the GGAs. The most common implementation of GGA is the PBE functional.^32^ A more recent class of functionals are known as *meta*-GGA functionals, which additionally include the second derivative of the local density.^33^ An implementation of this is the rSCAN functional.^34^ The mean-field approximation introduces a self-interaction error that leads to a delocalization of electrons that is not physical. A way to circumvent this is to introduce a *U* term that penalizes partial occupations. The *U* parameter can be found empirically by fitting to electronic band structures or can be self-consistently derived.^35^

Spin-polarized single-point energy SCF calculations, geometry optimizations, core loss spectral calculations, and electronic densities of states were obtained using the CASTEP plane-wave DFT code^36^ version 20.11, using default on-the-fly (OTF) ultrasoft pseudopotentials. The exchange correlation functionals PBE and rSCAN are used, and the impact of the Hubbard U is investigated by adding a *U* to PBE electronic structure calculations (hereafter referred to as PBE + *U*). The OTF ultrasoft pseudopotentials are at the GGA level for PBE and PBE + *U* and are at the *meta*-GGA level for rSCAN. The atomic positions and cell vectors were optimized using both the PBE and rSCAN functionals, and PBE geometry optimized structures were used for PBE + *U* electronic structure calculations. *U* values for the TM atomic d orbitals were based on literature values and were 5 eV for LiCoO_2_,^37^ 6 eV for LiNiO_2_,^25,38^ and 4.5 eV for *m*-LiMnO_2_.^39^ Simulated XAS spectra were obtained using optados,^40^ using the adaptive broadening scheme. The spectra were then convoluted with a Gaussian function comprising a fixed width of 0.5 eV, which was used as an approximation of the experimental resolution.^41^ Although the resolution of the beamline was higher than this (see Experimental Methods section), it was found that 0.5 eV produced spectra more suitable for comparison with experimental data. The spectra were also convoluted with a Lorentzian with a fixed width of 0.14 eV, which was used to represent the lifetime of the initial state, the value of which was obtained from the measurements carried out by Campbell and Papp.^42^ Finally, the spectra were convoluted with an energy-dependent Lorentzian scale, used to represent the lifetime of the final state, which has a value of 0.1(*E* – *E*_Fermi_) eV, the default optados value, which was also the best match to the experimental results. The spectra that were convoluted to include lifetime and instrumentation broadening effects are hereafter called broadened spectra, and those which were not convoluted to include lifetime and instrumentation broadening effects are referred to as unbroadened spectra. The convergence protocol is given in the Supporting Information and converged values of numerical parameters, used across different materials and exchange–correlation functionals are given in Table S1.

Core-hole calculations were conducted by modifying the pseudopotential definition and removing a 1s electron from the chosen oxygen site. A background charge was included to charge compensate for the core hole. Supercells were used to prevent neighboring core-holes from interacting with each other. The specific sizes used are given in Table S1 in the Supporting Information. The only system in this study with two symmetrically inequivalent oxygen sites is *m*-LiMnO_2_ with antiferromagnetic (AFM) magnetic ordering. In this case, two core-hole calculations were conducted, and then the spectra were added together using the method developed by Mizoguchi et al.^43^

To better interpret the origins of different spectral features in the experimental XAS, the projected density of states (pDOS) including a core-hole was calculated using CASTEP and processed using optados. For a closer analysis of the origin of certain spectral features, charge-density distributions were extracted using c2x^44^ and visualized using isosurfaces produced with VESTA.^45^ The charge density distributions shown herein were selected as representative after sampling. Sampling was carried out by obtaining the intensities of the overlap matrix for all combinations of *k*-point and band numbers and using c2x to extract charge-density distributions from the CASTEP check file at the *k*-point and band numbers with the highest overlap matrix intensities. The selection of combinations of *k*-point and band numbers in this manner was conducted within specific energy ranges corresponding to spectral features identified in unbroadened spin-up and spin-down core-loss spectra. The majority spin carrier within the simulation cell is labeled as spin up.

## Results and Discussion

### Comparison of DFT Functionals

**Figure 1:**
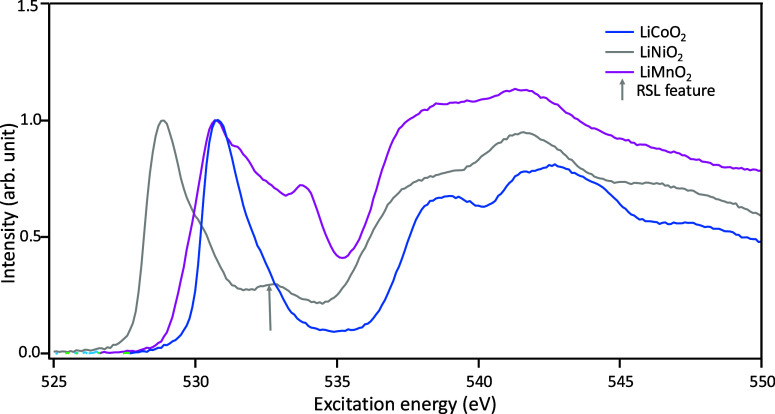
O K-edge FY experimental data of Li TM oxides considered in this study: LiCoO_2_, LiNiO_2_, and LiMnO_2_. The reduced surface layer feature present in LiNiO_2_ is highlighted with an arrow in the figure and is shown as a feature to exclude from interpretation. The spectra are normalized to the pre-edge peak.

O K-edge FY-XAS experimental spectra of the layered Li TM oxides—LiCoO_2_, LiNiO_2_, and *m*-LiMnO_2_—are presented in Figure 1. A pre-edge feature is seen between 530 and 534 eV for LiCoO_2_, 528–535 eV for *m*-LiMnO_2_, and 527–532 eV for LiNiO_2_. The feature centered at ∼532.5 eV in the LiNiO_2_ spectrum is attributable to a NiO-like reduced surface layer (RSL), as NiO gives a peak at this value and is thus excluded from interpretation.^46^ The main edge features are seen above 535 eV for all materials.

**Figure 2:**
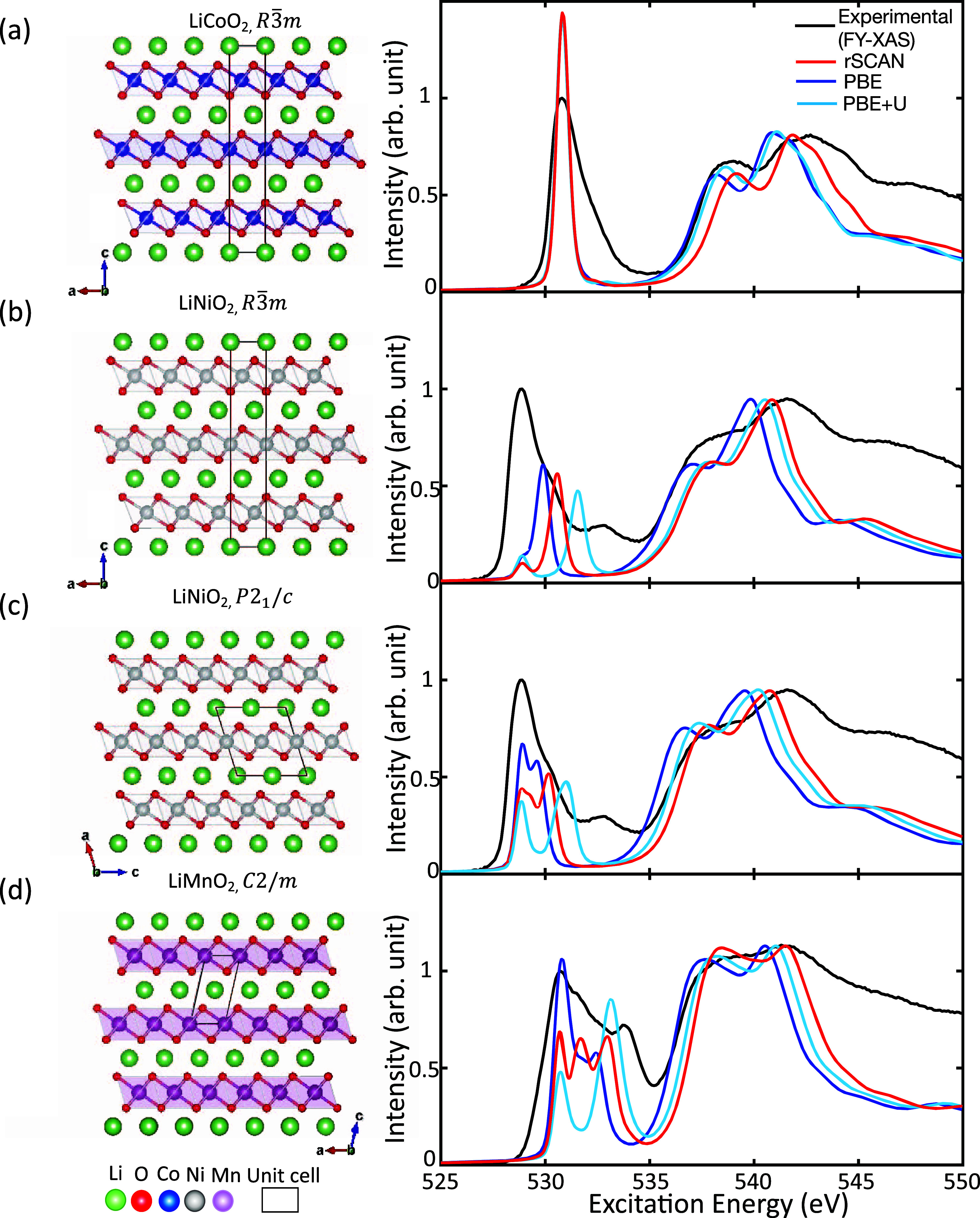
Comparing simulated core loss spectra to experimental O K-edge FY-XAS for (a) *R*3̅*m* LiCoO_2_, (b) *R*3̅*m* LiNiO_2_, (c) *P*2_1_/*c* LiNiO_2_, and (d) *C*2/*m* LiMnO_2_.

The suitability of different DFT functionals for the interpretation of the experimental spectra is assessed to lay the foundation for atomistic interpretation of the O K-edge XAS of layered Li TM oxides. The structures used for theoretical core-loss calculations for comparison with experimental XAS are shown in Figure 2: LiCoO_2_ with *R*3̅*m* symmetry, two LiNiO_2_ structures with *R*3̅*m* and *P*2_1_/*c* symmetry, and LiMnO_2_ with *C*2/*m* symmetry. The initial structures were obtained from the inorganic crystal structure database (ICSD) for *R*3̅*m* LiCoO_2_,^47^*R*3̅*m* LiNiO_2_^48^ and *C*2/*m* LiMnO_2_.^27^*P*2_1_/*c* LiNiO_2_ is a theoretically predicted structure and was calculated by Das et al.^49^ Co was modeled as low-spin Co^3+^,^50^ Ni as low-spin Ni^3+^,^51,52^ and Mn as high-spin Mn^3+^.^27^ Both LiNiO_2_ structures were modeled with a FM magnetic configuration, as Chakraborty et al.^51^ and Chen et al.^53^ showed using DFT that the FM configuration is lower in energy than the AFM configuration for the *R*3̅*m* structure and is minimally different for the JT-distorted structures. The LiMnO_2_ structure was modeled with an AFM magnetic configuration, as magnetization studies^54,55^ show that the Neél temperatures is ∼250 K, and this is corroborated by theoretical calculations conducted by Banerjee et al.^39^

Although X-ray diffraction (XRD) is consistent with LiNiO_2_ exhibiting *R*3̅*m* symmetry,^56^ extended X-ray absorption fine structure (EXAFS),^57^ and neutron diffraction measurements^58^ suggest the presence of local distortion.^57^ Sicolo et al.^59^ proposed that there is a dynamic JT effect and that due to the different length scales probed by XRD and EXAFS, EXAFS probes locally such that the temporal variation in the long bond direction does not average out. The DFT calculations conducted in their article show that the lowest-energy structure is LiNiO_2_ in a *P*2_1_/*c* symmetry. Thus, the spectra arising from both *R*3̅*m* and *P*2_1_/*c* LiNiO_2_ structures have been included to compare with the experimental data. To highlight the presence and absence of the JT distortion, the LiNiO_2_ structure with *R*3̅*m* symmetry is also referred to as undistorted LiNiO_2_, and the LiNiO_2_ structure with *P*2_1_/*c* symmetry is referred to as noncollinear JT distorted LiNiO_2_.

The theoretical spectra generated by using each of the three exchange–correlation functionals selected in this study are compared to experimental FY-XAS data. Figure 2 depicts the comparison of experimental FY-XAS (black) with the core-loss spectra calculated using PBE (dark blue), PBE + *U* (light blue), and rSCAN (red). To compare the broadened theoretical spectra with the experimental XAS, the pre-edges of the broadened calculated spectra have been aligned with the corresponding pre-edges of the experimental data. The calculated spectra were normalized to match the maximum intensity in the main-edge region of the experimental spectra. The experimental peak intensities are interpreted with the caveat that the presence of surface layers (potentially up to tens of nm in thickness) such as the RSL may affect the relative peak intensities since the probing depth of FY-XAS is only ∼200 nm.^12^

For all materials considered in this study, the core-loss spectra calculated using the rSCAN functional consistently capture the experimental peak positions for features in the main edge region better than those calculated with PBE or PBE + *U*. This is especially evident in LiCoO_2_. The results are more nuanced when the pre-edge contains multiple peaks. The pre-edge spectral shapes calculated with PBE, PBE + *U* and rSCAN for the *R*3̅*m* LiNiO_2_ structure do not show a good match with the experimental spectrum, see Figure 2b. The spectral shape of the pre-edges calculated for the noncollinear JT distorted LiNiO_2_ with PBE and rSCAN both show much better matches, while that calculated by PBE + *U* still does not capture the experimental pre-edge spectral shape well, Figure 2c. As we discuss in a later section, the inclusion of the JT distortion in the structure of LiNiO_2_ and the better match of the resulting DFT-calculated core-hole core-loss spectra does not necessarily confirm the experimental structure of LiNiO_2_.

The spectral shape of the pre-edges calculated for the *m*-LiMnO_2_ with PBE and rSCAN both show much better matches, while that calculated by PBE + *U* still does not capture the experimental pre-edge spectral shape well, as depicted in Figure 2d. rSCAN provides the closest match to the experimental peak positions, whereas PBE captures the relative peak intensity of the experimental pre-edge the best of the three functionals used but does not capture the peak positions.

The O p component of the pDOS of the atom containing the core-hole and the angular-momentum channel resolved pDOS of its nearest neighbors (as depicted in Figures S1–S5), along with consideration of the allowed spectroscopic transitions, inform the interpretation of the spectra. Consistent with prior literature,^21,51^ the pDOS of core-hole spectral calculations shows that the pre-edge features correspond to an energy range containing TM-d states and O p states, with their very similar shapes indicating they are strongly hybridized with each other. The region above 535 eV corresponds to the O p, Li-s, and TM-s,p states in the pDOS, with the similar shapes of the O p and TM-s,p states again indicating strong hybridization. Comparisons across the different functionals confirm that rSCAN better captures the energy scale, as the states seem to be more spread across the energy range. The shape of the O p states modeled by rSCAN also closely resembles that modeled by PBE, the only difference being the energy spacing between features.

Compared to modeling spectra with PBE + *U* and PBE, the rSCAN functional strikes a good compromise between capturing O K-edge peak positions and spectral features. The semi-local effect of rSCAN, linked to inclusion of the second derivative of the electron density in the construction of the functional, leads to the spreading out of states across the whole energy scale, giving better agreement with experimental peak positions. This result potentially indicates that a direction to consider for functional development for the purpose of O K-edge spectral DFT-modeling is to construct functionals that are more sensitive to semi-local effects. PBE + *U* is less effective in this regard as the influence of the U on the O K-edge spectrum is more indirect, altering the TM-d states which are hybridized with the O p states. Disentangling the effects of rSCAN on geometric and electronic structures is shown in Figure S6. The discussion in the Supporting Information shows that even when using same structure, rSCAN leads to a stretching of states that leads to better modeling of the edge positions than PBE and PBE + *U*.

Having shown the general suitability of rSCAN and the limitations of PBE + *U* for interpreting the spectral features of layered Li TM oxides, our results herein suggest rSCAN is a favorable candidate for interpreting the spectra of materials with more complex oxygen environments (such as NMC materials) and at different stages of charge. This is consistent with the work of Chakraborty et al., in which the ground-state (without a core-hole) DOS of *R*3̅*m* LiCoO_2_, *R*3̅*m* LiNiO_2_, and orthorhombic LiMnO_2_ were modeled using SCAN, PBE, and PBE + *U*.^60^ Chakraborty et al. found that while the inclusion of a Hubbard U term in the Hamiltonian can improve the electronic band gap and voltage, it can distort the electronic structure by predicting a higher contribution of O p states near the Fermi level along with a significantly lower contribution from TM-d states, which they claim is contradictory to experimental XPS results.^61^ Furthermore, the dependence of the U value on the elements and their oxidation states limits its practical application for studying delithiation behavior (an important step toward understanding the redox mechanism). Due to its general suitability for modeling O K-edge XAS, rSCAN was therefore selected for subsequent analyses of spectral features in this article.

### Impact of Changes in the Oxygen Environment on O K-Edge XAS Spectral Features

**Figure 3:**
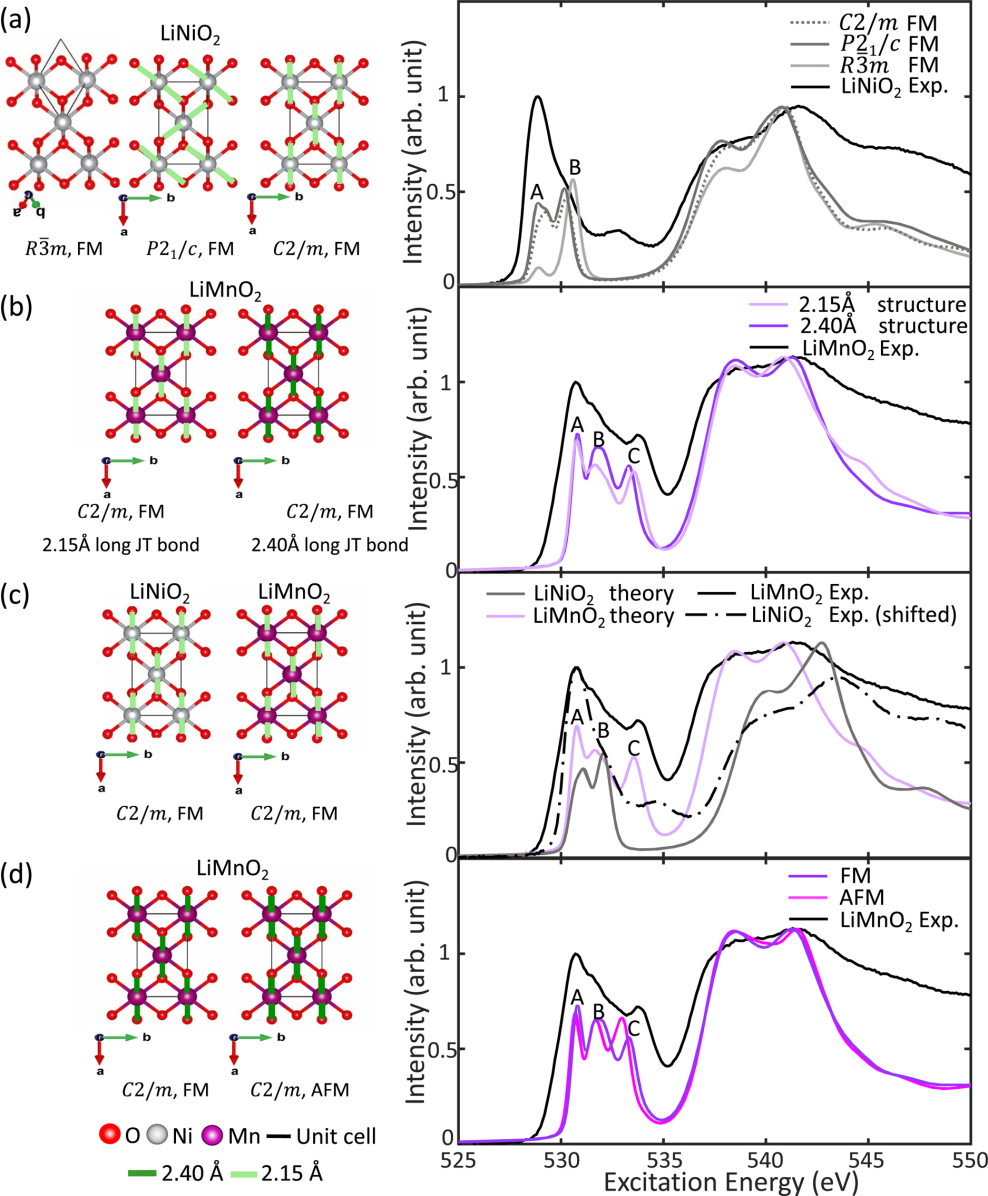
Impact of (a,b) varying the degree of JT distortion, (c) identity of the TM species, and (d) magnetism on the spectral features of the O K-edge XAS (labeled Exp. in graph legend) is depicted. Structures are, from left to right in each row: (a) LiNiO_2_ with no JT distortion, noncollinear JT distortion, and collinear JT distortion; (b) LiMnO_2_ and FM, but vary in structure; the right-hand structure has a larger JT distortion than the left; (c) LiNiO_2_ and LiMnO_2_, both FM and structurally identical, but vary in TM identity; (d) LiMnO_2_ and structurally identical, but the left-hand structure is FM and the right-hand is AFM.

Investigating whether DFT-calculated spectra are sufficiently sensitive to subtle changes in the oxygen environment is an important step toward atomistic interpretation of the layered Li TM oxides. To do this, a series of structures were selected to be systematically investigated as to the impact of changes in geometry, chemical species, and magnetism on the O K-edge spectra, as depicted in Figure 3. We focus on the shape of the pre-edge, which is of particular interest for understanding redox reactions, as it contains information about the hybridized TM-d and the O p orbitals.

### Impact of JT Distortion on Spectral Features

Three LiNiO_2_ structures were selected to investigate the impact of the degree and direction of JT distortion on the spectral features. Undistorted LiNiO_2_ with *R*3̅*m* symmetry, noncollinearly JT distorted LiNiO_2_ with *P*2_1_/*c* symmetry, and collinearly JT distorted LiNiO_2_ with *C*2/*m* symmetry are depicted in Figure 3a. The structures of undistorted and noncollinearly JT distorted LiNiO_2_ are the same as those discussed in the previous section; the collinear JT LiNiO_2_ model was made by substituting sodium with lithium atoms in an initial NaNiO_2_ structure obtained from the ICSD^27,39,62^ and relaxing the structure according to the parameters described in the computational details section. Again, all of the LiNiO_2_ structures were modeled with an FM magnetic configuration.

The pre-edge of all the LiNiO_2_ theoretical spectra contain two subpeaks A and B, at lower and higher excitation energy respectively, shown in Figure 3a. Peak A significantly increases in intensity from the spectrum of the undistorted LiNiO_2_ structure to that of the JT distorted LiNiO_2_ structures. Peaks A and B look similar for both of the JT distorted structures, and they both are closer matches to the experimental spectral shape than the spectrum of the undistorted structure. All three calculated spectra predict similar positions for peak B. Thus, the pre-edge shape is most impacted by the inclusion of the JT distortion rather than the direction of the distortion.

**Figure 4:**
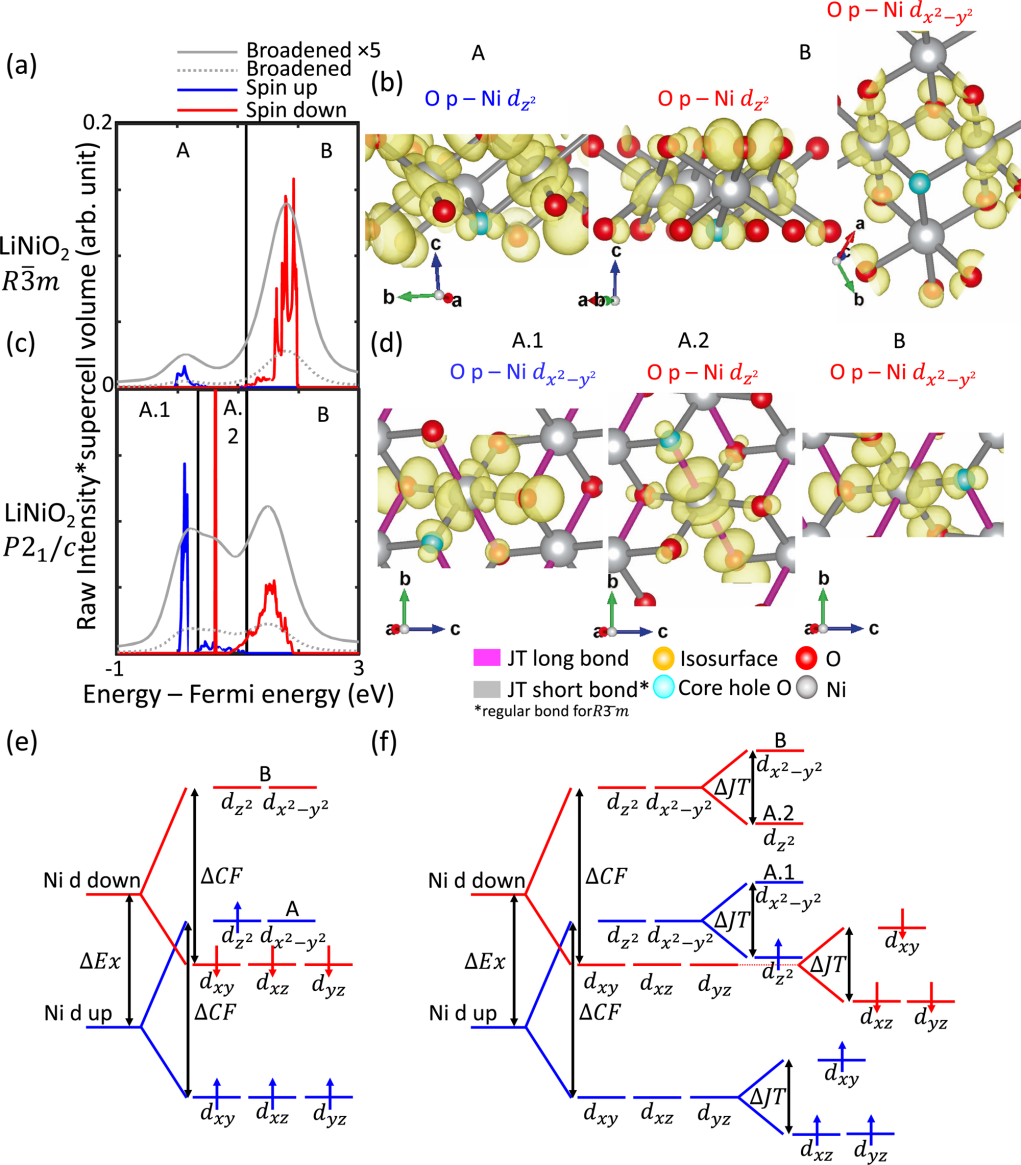
Origins of spectral features arising from JT distortions. Broadened and spin-resolved unbroadened spectra of (a) undistorted LiNiO_2_ and (b) noncollinearly JT distorted LiNiO_2_ are depicted. Representative charge-density distributions corresponding to key spectral features arising from (c) undistorted LiNiO_2_ and (d) noncollinearly JT distorted LiNiO_2_ are depicted. Schematic CFT diagrams are depicted for neighboring Ni in (e) undistorted LiNiO_2_ and (f) noncollinearly JT distorted LiNiO_2_.

To better understand the effect of JT distortion on the pre-edge features, the broadened spectra are compared with their respective spin up and spin down unbroadened spectra. The unbroadened spectral features are used as a reference to extract charge-density distributions, as explained in the Methods section, of which representative figures are depicted in Figure 4. The unbroadened and broadened spectra are aligned such that the Fermi energy is set to 0. To compare unbroadened spectra arising from calculations using different supercell sizes, the raw intensity output from optados is multiplied by the volume of the supercell as the overall intensity is scaled by the supercell volume. To better observe how the unbroadened spectral features correspond to those of the broadened spectra, the intensities of the broadened spectra are further multiplied by a factor of 5. We also use charge-density distributions to better understand the origins of pre-edge features within the framework of crystal-field theory (CFT). This analysis can qualitatively show how the effects of exchange splitting (ΔEx), crystal-field splitting (ΔCF), and the splitting associated with JT distortion (ΔJT) give rise to the arrangement of O p—TM-d states and, consequently, affect the spectral shape and features of the pre-edge of the O K-edge of layered Li TM oxides.

The origins of the pre-edge features for undistorted and noncollinear JT distorted LiNiO_2_ are examined, with the latter also considered representative of the collinear JT distorted structure given the minimal differences between their spectra. The broadened spectrum of the undistorted structure in Figure 4a shows a low intensity spin-up feature labeled A, and a much stronger spin-down feature labeled B. The corresponding charge-density distributions shown in Figure 4b, show the A and B features arise from transitions into states resembling O p hybridized to neighboring Ni d_*z*_^2^ orbitals and O p hybridized to neighboring Ni- orbitals. In contrast, the broadened spectrum of the JT distorted structure in Figure 4c shows much more sharply intense spin-up and spin-down features labeled A.1 and A.2 respectively. Representative charge-density distributions in Figure 4d reveal that the first spin-up feature A.1 in the unbroadened spectrum of the JT distorted structure arises from transitions into states strongly resembling O p hybridized with neighboring Ni orbitals. The sharply intense spin-down feature A.2 arises from transitions into states strongly resembling hybridized O p with neighboring Ni d_*z*_^2^ orbitals, and the spin-down feature B arises from transitions into states strongly resembling hybridized O p with neighboring Ni  orbitals. This distinct sequence of hybridized states observed in the JT distorted structures is absent for the undistorted structure.

The schematic diagrams of the neighboring Ni d states for the undistorted and JT distorted LiNiO_2_, depicted in Figure 4e,f, show how the overlapping effects of ΔEx, ΔCF, and ΔJT can result in a distinct sequence of hybridized states in the case of JT distorted LiNiO_2_. In the case of low spin Ni^3^+, ΔCF is such that electrons fill the spin-up and spin-down d_*xz*_, d_*yz*_ and d_*xy*_ states first before filling the d_*z*_^2^ and  states. In the case of the undistorted structure, as both d_*z*_^2^ and  states are degenerate, it is possible to see a combination of hybridized states without a preferential sequence of filling. In the case of JT-distorted structures, ΔJT divides the states such that d_*z*_^2^ is lower in energy than . Due to ΔEx, the lowest energy unoccupied Ni state hybridized to O p will be spin-up ; hence, we see in the charge-density distributions this very hybridized state corresponding to the first spin-up feature A.1, followed by the spin-down feature A.2 arising from a transition into a hybridized O p and neighboring Ni d_*z*_^2^ state.

Although including a JT distortion in the structure of LiNiO_2_ provides a pre-edge spectral shape that approaches that of the experimental data, in the case of LiNiO_2_ this analysis is not sufficient to conclusively confirm the structure giving rise to experimental XAS of LiNiO_2_. At the DFT-level, multiple models have been suggested to capture the local distortion indicated by EXAFS^63^ and neutron PDF;^58^ an example is a dynamic spin-disproportionated model^12,64^ in which formally Ni^2+^, Ni^3+^, and Ni^4+^ centers coexist. Additionally, at the DFT-level *R*3̅*m* LiNiO_2_ is predicted to be half-metallic.^51,65^ This is counter to experiment^66−68^ and dynamical mean-field theory calculations,^65^ both of which report a small band gap. Furthermore, the presence of defects in the sample could also contribute to the spectral shapes; it is known to be difficult to create purely stoichiometric LiNiO_2_, with even samples produced under well controlled conditions showing 1–2% Ni excess that occupies Li sites (Ni_Li_ defects).^69^ Lin et al. show that the electronic structure of ground state *R*3̅*m* LiNiO_2_ is altered by the presence of such Ni_Li_ defects;^70^ thus it is reasonable to expect that the core-hole spectra could also be altered.

Additionally, treating the electron–hole excitations via the Bethe–Salpeter equation (BSE) could be a way of improving the spectral shape of the pre-edge of LiNiO_2_ as it has been shown to increase peak intensity at the edge onset for a variety of materials^71,72^ by taking into account the effect of excitons. However, Ruiz et al.^22^ show minimal differences between DFT-calculated spectra and those calculated using BSE for the O K-edge XAS of LiCoO_2_. It is unclear from the reported literature whether this would also be true for the O K-edge XAS of LiNiO_2_. It would also be difficult to disentangle the effects of heterogeneity arising from a range of possible structure models and defects using BSE, which is known to be a computationally expensive method.^73^ Due to this, we limit further discussion to how to extend DFT-based modeling to accommodate heterogeneity.

Assessing the wide range of structure models and defects proposed for LiNiO_2_ and their influence on the O K-edge is beyond the scope of this work. Rather, the goal of this work is to assess the impact of varying the DFT exchange correlational functional used and key structural, chemical, and magnetic features of the coordinating environment on the DFT-calculated spectral features of layered Li TM oxides.

The impact of the JT distortion is further investigated by comparing the spectra arising from the *m*-LiMnO_2_ structures depicted in Figure 3b. Both structures include collinear JT distortions, with the first structure isostructural to collinear JT LiNiO_2_, and the latter structure isostructural to experimentally measured *m*-LiMnO_2_, with a larger degree of JT distortion and thus monoclinicity. Both have been modeled with the same magnetic configuration (FM) to isolate the impact of the JT distortion. The calculated spectra arising from these two *m*-LiMnO_2_ structures are compared in Figure 3b, along with the experimental XAS of *m*-LiMnO_2_. There are noticeable, albeit subtler, changes in the spectral shape of the pre-edge as the JT distortion is increased; the increase in JT distortion creates a more pronounced peak B and lowers the separation between peaks B and C. Interestingly, the *m*-LiMnO_2_ structure with less JT distortion shows a closer match between the energy position of peak C and the experimental position (around 534 eV). Given that the differences in the spectra between the *m*-LiMnO_2_ structures are much less drastic than those seen between the undistorted and distorted LiNiO_2_ structures, further analysis using charge-density distributions is deemed unnecessary.

### Changing Transition Metal

Investigating the impact on the K-edge spectra of the TM as the TM ratio is varied in NMC materials requires a solid atomistic interpretation of the spectra in the parent materials. In particular, being able to compare the spectra arising from identical geometric structures comprising the same magnetic configuration, with the only difference being the identity of TM, will highlight the impact that the identity of the TM species has on the spectral features of the O K-edge XAS. While the ground state structure of LiNiO_2_ is debated, the structural models used in this section still represent some of the possible oxygen environments that occur in NMC materials. Thus, it is useful to compare DFT-calculated spectra arising from these structures to assess whether the differences between the DFT-calculated spectra are similar to those observed in experimental XAS.

For this purpose, Figure 3c compares the spectrum of collinearly JT distorted LiNiO_2_ to that of an isostructural *m*-LiMnO_2_, a theoretical structure that is constructed by replacing the Ni in collinearly JT distorted LiNiO_2_ with Mn. The calculated spectra are compared to those of the experimental LiNiO_2_ XAS, which has been shifted so that the pre-edge aligns with that of the experimental *m*-LiMnO_2_, which is also depicted. The pre-edge of the spectrum arising from the *m*-LiMnO_2_ structure contains an additional peak, labeled C. This matches the trend observed in the experimental data. Additionally, the differences in peak positions of spectral features in the main edge region of calculated spectra correspond reasonably well with the experimental spectra.

**Figure 5:**
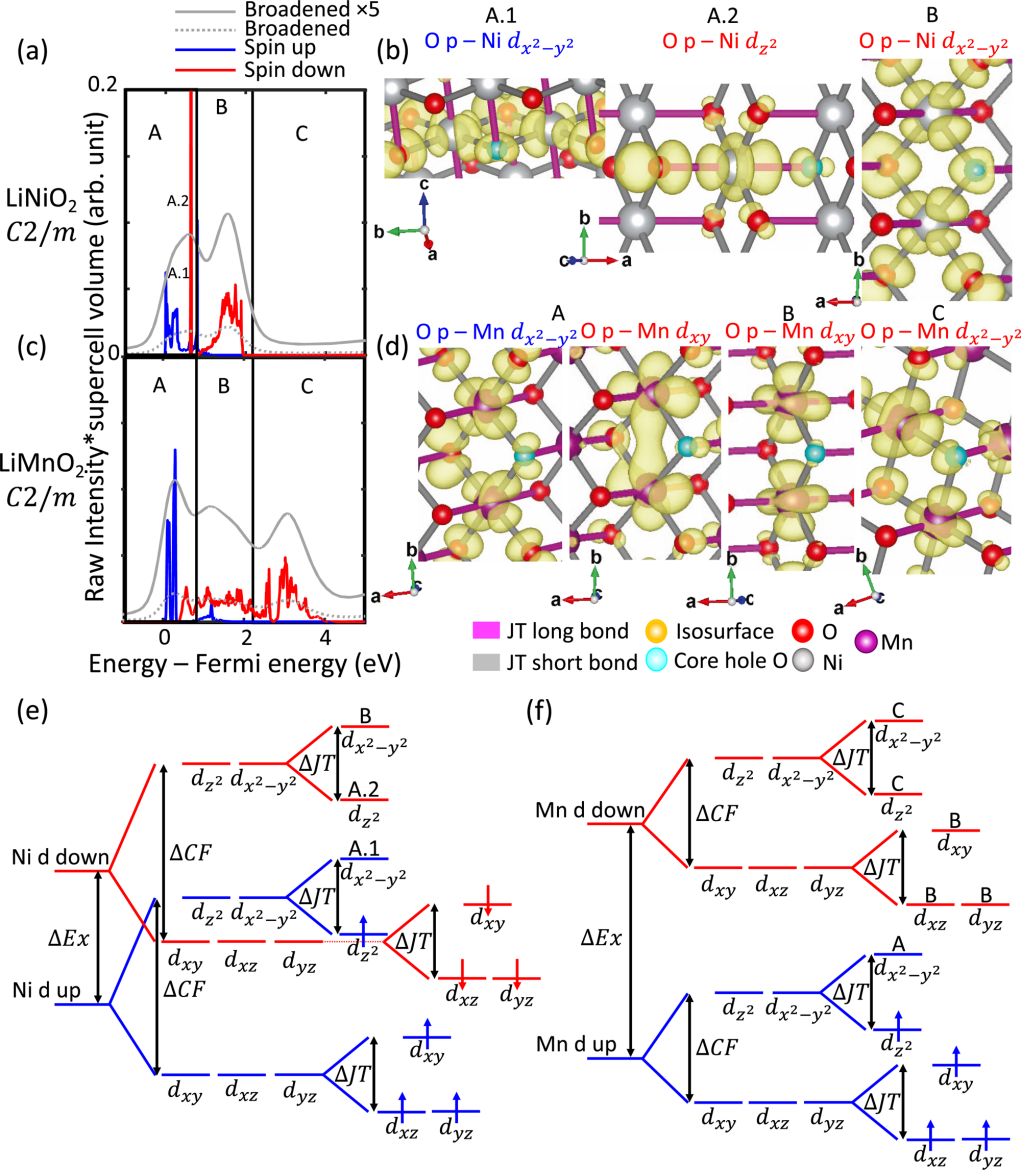
Origins of spectral features arising from different neighboring TMs. Broadened and spin-resolved unbroadened spectra of (a) collinearly distorted LiNiO_2_ and (b) isostructural *m*-LiMnO_2_ are depicted. Representative charge-density distributions corresponding to key spectral features arising from (c) collinearly distorted LiNiO_2_ and (d) isostructural *m*-LiMnO_2_ are depicted. Schematic CFT diagrams are depicted for (e) the neighboring Ni in LiNiO_2_ and (f) the neighboring Mn in LiMnO_2_.

Due to drastic differences in pre-edge spectral features when the TM metal identity is changed, we again consider charge-density distributions to understand the origins of the different pre-edge features (see Figure 5). The spectra arising from collinear JT LiNiO_2_ and isostructural *m*-LiMnO_2_ are shown in Figure 5a,c. Feature A of both spectra shows significant contributions from spin-up states. Representative charge-density distributions (Figure 5b,d) show these spin-up states correspond to transitions into O p hybridized to neighboring TM  orbitals. In the LiNiO_2_ spectrum, feature A also arises from a sharply intense spin-down feature, which corresponds to a transition into O p hybridized to neighboring Ni d_*z*_^2^ states. Feature B in both spectra is dominated by spin-down contributions. For the LiNiO_2_ spectrum, charge-density distributions reveal that feature B predominantly arises from transitions into states resembling O p hybridized to neighboring Ni. For the *m*-LiMnO_2_ spectrum, charge-density distributions reveal that feature B corresponds to transitions to O p hybridized to neighboring Mn d_*xy*_, Mn d_*xz*_, and Mn d_*yz*_ states. Finally, feature C, which only arises for the *m*-LiMnO_2_ spectrum, is shown to arise predominantly from transitions into states that somewhat resemble hybridized O p and Mn , and hybridized O p and Mn d_*z*_^2^.

A schematic diagram indicating the arrangement of Ni and Mn d states for both structures is shown in Figure 5e,f. As ΔEx is proportional to the number of unpaired electrons present,^73^ it is estimated that Mn^3+^ (4 unpaired electrons) has a larger ΔEx than Ni^3+^ (1 unpaired electron). Thus, the lowest-energy unoccupied state is an O p hybridized to a neighboring spin-up Mn  state. Due to the presence of additional unoccupied spin-down Mn d states compared to Ni, this results in the presence of the additional peak C. Thus, the differences in the number of unpaired electrons between Ni^3+^ and Mn^3+^ result in not only different hybridized states but also a difference in the arrangement of these states. Thus, DFT-calculated spectra are sensitive to changes in neighboring TM identity and could be used to atomistically interpret O K-edge XAS arising from NMC materials with different ratios of TM.

### Changing Magnetic Configuration

The magnetic configuration changes at different states of charge and at different TM ratios in NMC materials. Assessing whether DFT-calculated spectra are sensitive to the influence of magnetic configuration is thus an important step toward the atomistic interpretation of more complex environments.

Figure 3d compares the calculated spectra O K-edge arising from *m*-LiMnO_2_ modeled in an AFM configuration with that in an FM configuration. The changes in spectral shape are subtle for this comparison, with both spectra having very similarly shaped pre-edge spectral features A and B. As the changes are subtle, further analysis with charge-density distributions is not conducted. The structure that shows a slightly closer match with the position of peak C in the experimental spectrum is the FM rather than AFM, even though the former has a lesser degree of JT distortion and monoclinicity than the experimental structure, and it is the latter that has the experimental structure parameter and gives a slightly lower energy structure with the AFM configuration.^39^ Banerjee et al. show that even subtle changes to the Li-content of *m*-LiMnO_2_ can change the magnetic configuration from AFM to ferrimagnetic.^39^ It is also worth noting that the experimental XAS spectra are measured at room temperature, which is higher than the Neél temperature of ∼250 K; thus, there could be a saturation magnetization at room temperature that is different from ground-state 0 K AFM.

It is also worth considering whether there is heterogeneity in the structure of the experimentally measured *m*-LiMnO_2_ as another source for the slight differences between the theoretical and experimental spectra, and to this end, Figure S7 shows XRD and scanning transmission electron microscopy measurements. Rietveld refinements of the XRD patterns (Figures S7a,b) indicate that ion exchange is successful and that the final phase is indeed *m*-LiMnO_2_. Additionally, annular dark field images, visualized along the [100] zone axis, show that there is negligible cation mixing (see Figure S7d). However, some twin boundaries are observed (see Figure S7e) as is typically expected from ion-exchange synthesis.^74^ It is unclear what impact the stacking faults may have on the O K-edge spectral features, but it may account for some of the small differences between the calculated spectra and the data. Further exploring how twin boundaries impact the spectral features could be the focus of future work.

## Conclusions

In summary, this work provides a thorough evaluation of the application of DFT to atomistically interpret the experimental K-edge spectra of layered Li TM oxides used as battery cathode materials. Although some examples of the comparison between first-principles and experimental core-loss spectra for these materials exist, this has not previously been carried out systematically across a variety of different exchange–correlation functionals. By assessing the suitability of different DFT functionals, we show the extent to which DFT-calculated spectra can be used for the atomistic interpretation of the O K-edge spectra of the Li TM oxides. DFT modeling of the O K-edge core loss spectra of LiCoO_2_, and monoclinic *m*-LiMnO_2_ closely reproduces the features present in experimental XAS of these materials. The semi-local *meta*-GGA functional rSCAN better reproduces the relative energy positions of spectral features compared with the GGA functional PBE and PBE with the DFT + *U* method. Careful analysis of the origins of pre-edge features shows that DFT-calculated spectra are also sensitive to geometric distortion, chemical species, and magnetic configuration. This analysis shows that the DFT-calculated pre-edge shape is most affected by a change in the identity of the neighboring TM and the inclusion of JT distortion in the LiNiO_2_ structure. Thus, the DFT-calculated pre-edge is sensitive not just to the chemical species of the coordinating environment but also to geometric distortion. The spectral shape of the pre-edge is more subtly, but still noticeably, affected by the degree of JT distortion and the magnetic ordering of the neighboring TM.

Direct comparison of experimental spectra with theoretical spectra arising from simple structural models highlights the influence of heterogeneity present even in pristine materials. The impact of heterogeneity on the spectral features, such as twin boundaries in *m*-LiMnO_2_ and possible spin-disproportionation and antisite defects in LiNiO_2_, is an interesting direction for further exploration. Understanding the influence of these local environments on the core loss spectrum likely requires the development of approaches that can identify key spectral fingerprints from more complex models of materials.^75,76^ Such methodology development could then be extended to observe the effect on the spectral shape at different states of charge or when changing the TM ratio to create different NMC ratios. Thus, this article and the future directions that it opens up show the extent to which DFT-calculated spectra can be used to interpret experimental data and determine the oxygen environments of layered Li-ion battery cathode materials.

## Data Availability

The data sets generated during this study are available from the ORA repository www.ora.ox.ac.uk, 10.5287/ora-o84me8rd9.

## Supporting Information Available

Details of the convergence protocol and converged computational parameters; core-hole pDOSs to understand DFT-calculated spectra used to compare to experimental XAS; effect of the rSCAN functional on electronic and geometric structure; and experimental characterization of LiMnO_2_ (PDF)
