# Supplementary material for: Atomistic Interpretation of the Oxygen K-Edge X-ray Absorption Spectra of Layered Li-Ion Battery Cathode Materials
Source: Chem Mater. 2024 Nov 12;36(22):11051–64. doi: 10.1021/acs.chemmater.4c01870 (PMC11603537; doi:10.1021/acs.chemmater.4c01870)
Supplement: Supplementary file 1 — cm4c01870_si_001.pdf [file cm4c01870_si_001.pdf]

Supplementary Information: An atomistic interpretation of the oxygen K-edge X-ray absorption spectra of layered Li-ion battery cathode materials

Namrata Ramesh,[†] Hrishit Banerjee,[‡,¶,§] Jack E. N. Swallow,[†,§] Erik Björklund,[†,§] Ava Dean,[∥] Pravin Didwal,[†,§] Michael Fraser,[†,§] Conor M. E. Phelan,[†] Lijin An,[†] Jasper Singh,[†] Jarrod Lewis,[†] Weixin Song,[†,§] Robert A. House,[†,§] Andrew J. Morris,[⊥,§] Robert S. Weatherup,[†,§] and Rebecca J. Nicholls[*,†]

†*Department of Materials, Oxford University, Oxford, OX1 3PH, UK*

‡*Yusuf Hamied Department of Chemistry, University of Cambridge, Cambridge, CB2 1EW, UK*

¶*School of Science and Engineering, University of Dundee, Scotland, DD1 4HN, UK*

§*The Faraday Institution, Quad One, Harwell Science and Innovation Campus, Didcot OX11 0RA, UK*

∥*Department of Physics, University of York, York, YO10 5DD, UK*

⊥*School of Metallurgy and Materials, University of Birmingham, Birmingham, B15 2SE, UK*

E-mail: rebecca.nicholls@materials.ox.ac.uk

Further information on computational methods

Table S1: Table of parameters. For simplicity of representation, only one set of structural parameters are shown, that from the initial structure before geometry optimization with different functionals. Supercell sizes are constructed from the relaxed cells. *: .cif filed use to construct is the $NaNiO_2$ with substituted Li. **: refers to all three $LiMnO_2$ structures presented in Figure 3b-d; the cut-off energy, k-point spacing and fine grid scale values used for these structures are the same. ***: for simplicity of representation's sake, showing the .cif file parameters of only one of the three structures: that of the experimentally measured structure from the ICSD. ****the supercell size is 2×3×2 for PBE and PBE+U.

Material	Symmetry	Cut off energy (eV)	k-point spacing (Å-1)	Spectral k-point spacing (Å-1)	Fine grid scale rSCAN	Fine grid scale PBE	U value (eV)	Conventional cell from CIF (x*y*z Å)	Supercell size
$LiCoO_2$	$R\bar{3}m$	850	0.03	0.02	1.75	1.75	5	2.81×2.81×14.03	2×2×1
$LiNiO_2$	$R\bar{3}m$	850	0.03	0.02	2	1.75	6	2.88×2.88×14.19	2×2×1
$LiNiO_2$	$P2_1/c$	850	0.03	0.02	3	1.75	6	4.97×2.92×4.89	2×3×2
$LiNiO_2$	$C2/m$	850	0.03	0.02	3	1.75	6	5.32×2.85×5.58*	2×3×2
m-$LiMnO_2$**	$C2/m$	1000	0.03	0.02	4	4	4.5	5.43×2.81×5.39***	2×4×2****

Single-point energy SCF calculations were conducted to converge the final energy with respect to Brillouin zone integration (k-point spacing), fine grid scale (which determines the maximum size of G-vectors), and plane-wave cut-off energy. Geometry optimisations were then performed to converge the structure parameters with respect to k-point spacing, fine grid scale, and plane wave cut-off energy. The structures were geometry optimised such that the forces were below 0.05 eV $Å^{-1}$. Finally, non core-hole broadened spectra generated by varying the geometry optimised structure used, k-point spacing, spectral k-point spacing, plane wave cut-off energy, and fine grid scale were compared to select a set of parameters that provided minimal differences between the spectra. The converged values of these numerical parameters, used across different materials and exchange-correlation functionals are given in Table S1 in the supplementary information (SI). Supercell sizes were selected such that there were approximately 10Å of distance between the neighboring core-holes in each direction. When a smaller supercell was used for the sake of computational efficiency, the broadened spectra was always compared to that obtained from a larger supercell to ensure that the features looked qualitatively similar.

As the systems are magnetic, whenever a core-hole was added with the default settings of density mixing parameters, it distorted the magnetic configuration in an unphysical manner. The issue was solved by defining the spin configuration in the CASTEP .cell file, and fixing the total spin such that it does not change between SCF iterations in the CASTEP .param file. There is not yet an option in CASTEP to fix the individual atomic moments; thus, the mixing parameters associated with charge and spin were altered to obtain a final magnetic configuration that was not unphysically altered by the core-hole.

pDOS of core-hole core-loss calculations

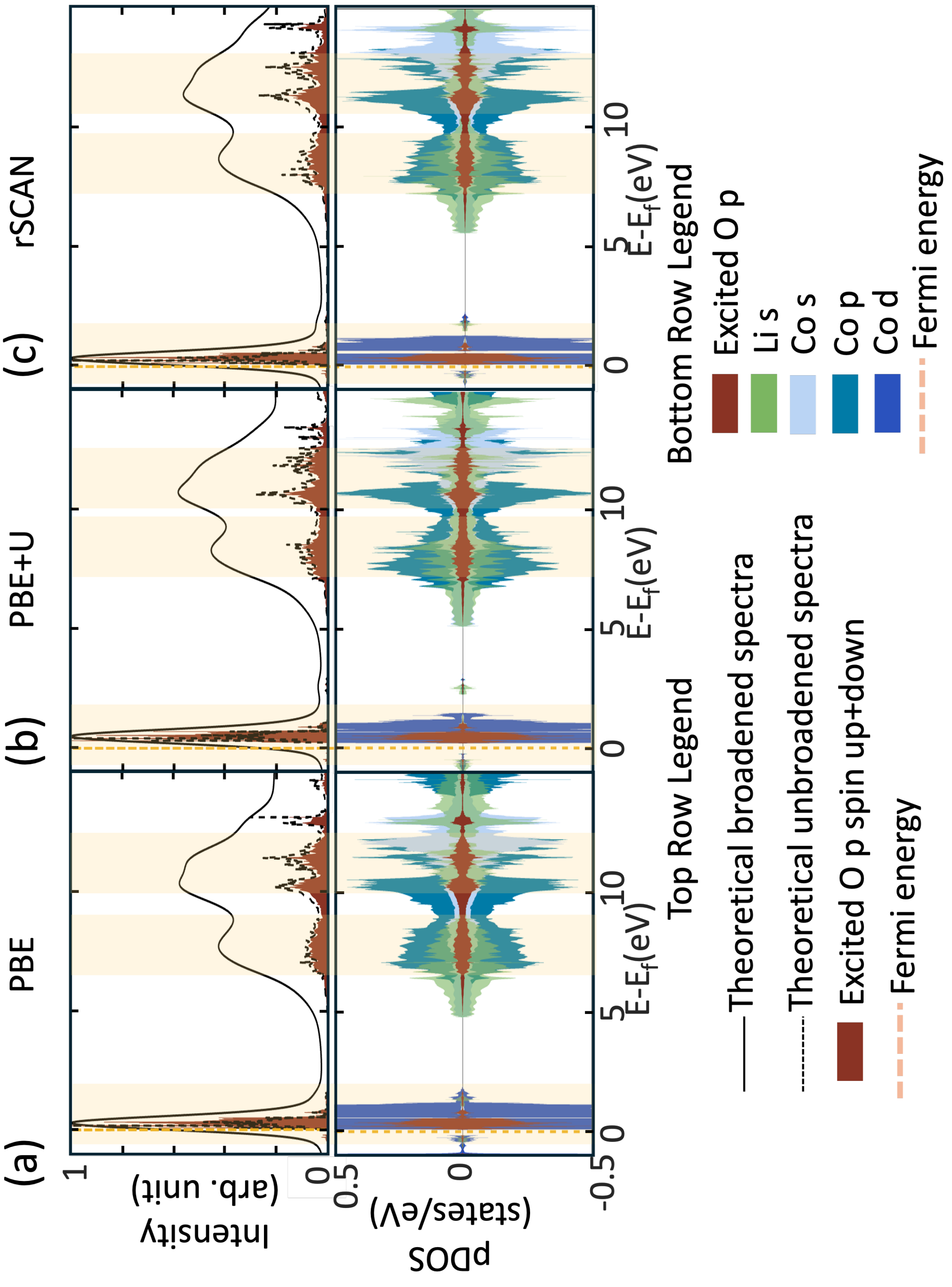

Figure S1: $LiCoO_2$ pDOS. (a) PBE (b) PBE+U (c) rSCAN. Row 1 is the broadened and unbroadened theoretical spectra compared to the sum of the spin-up and down O-p states of the excited O; Row 2 is the O s and p pDOS of the excited oxygen, along with the pDOS of nearest neighbor Li and Co. E_f is the Fermi energy.

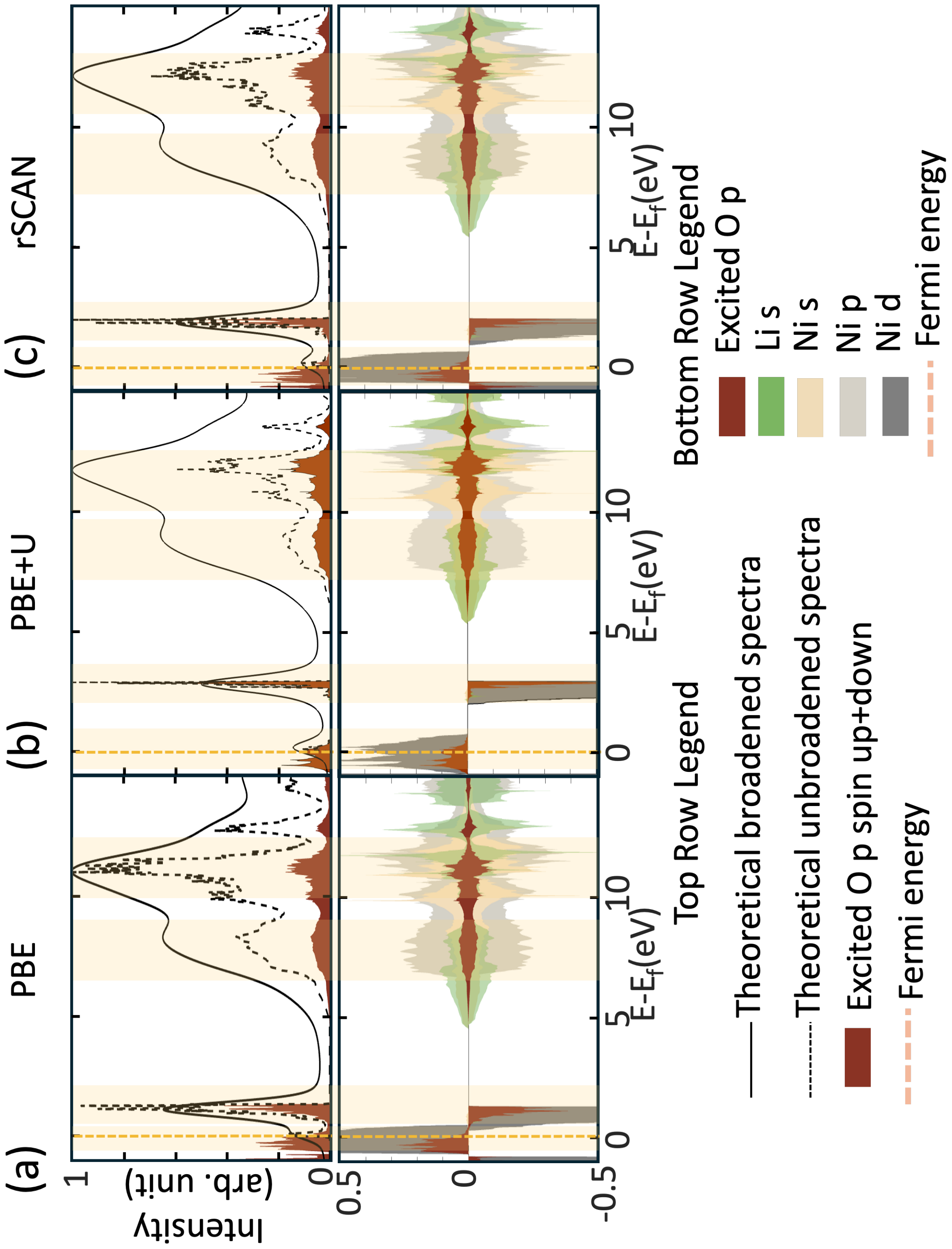

Figure S2: $LiNiO_2$ rhombahedral pDOS. (a) PBE (b) PBE+U (c) rSCAN. Row 1 is the broadened and unbroadened theoretical spectra compared to the sum of the spin-up and down O-p states of the excited O; Row 2 is the O s and p pDOS of the excited oxygen, along with the pDOS of nearest neighbor Li and Ni. E_f is the Fermi energy.

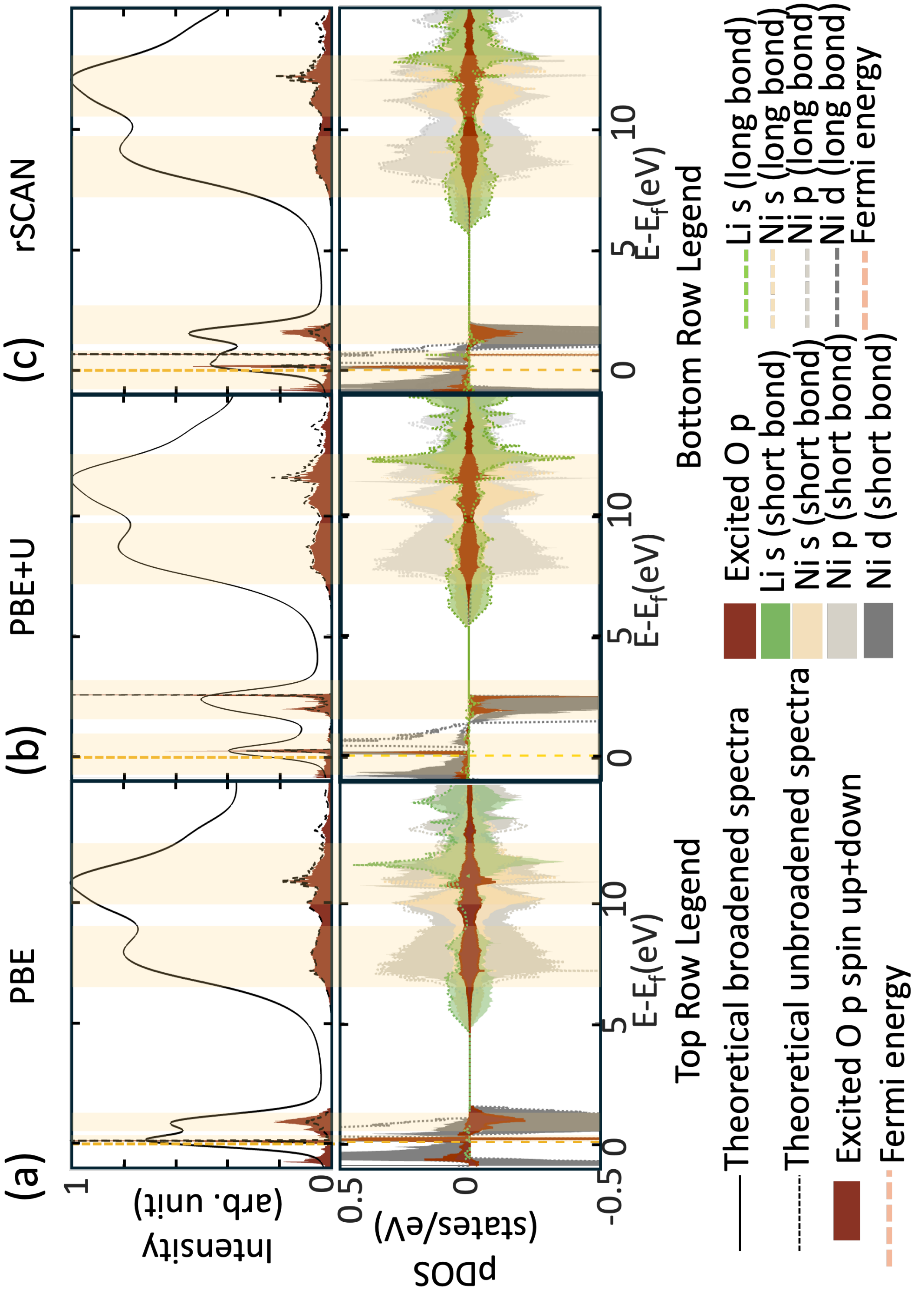

Figure S3: $LiNiO_2$ JT distorted pDOS with $P2_1/c$ symmetry. (a) PBE (b) PBE+U (c) rSCAN. Row 1 is the broadened and unbroadened theoretical spectra compared to the sum of the spin-up and down O-p states of the excited O; Row 2 is the O s and p pDOS of the excited oxygen, along with the pDOS of nearest neighbor Li and Ni (short JT bond) and the second nearest neighbor Li and Ni (long JT bond). E_f is the Fermi energy.

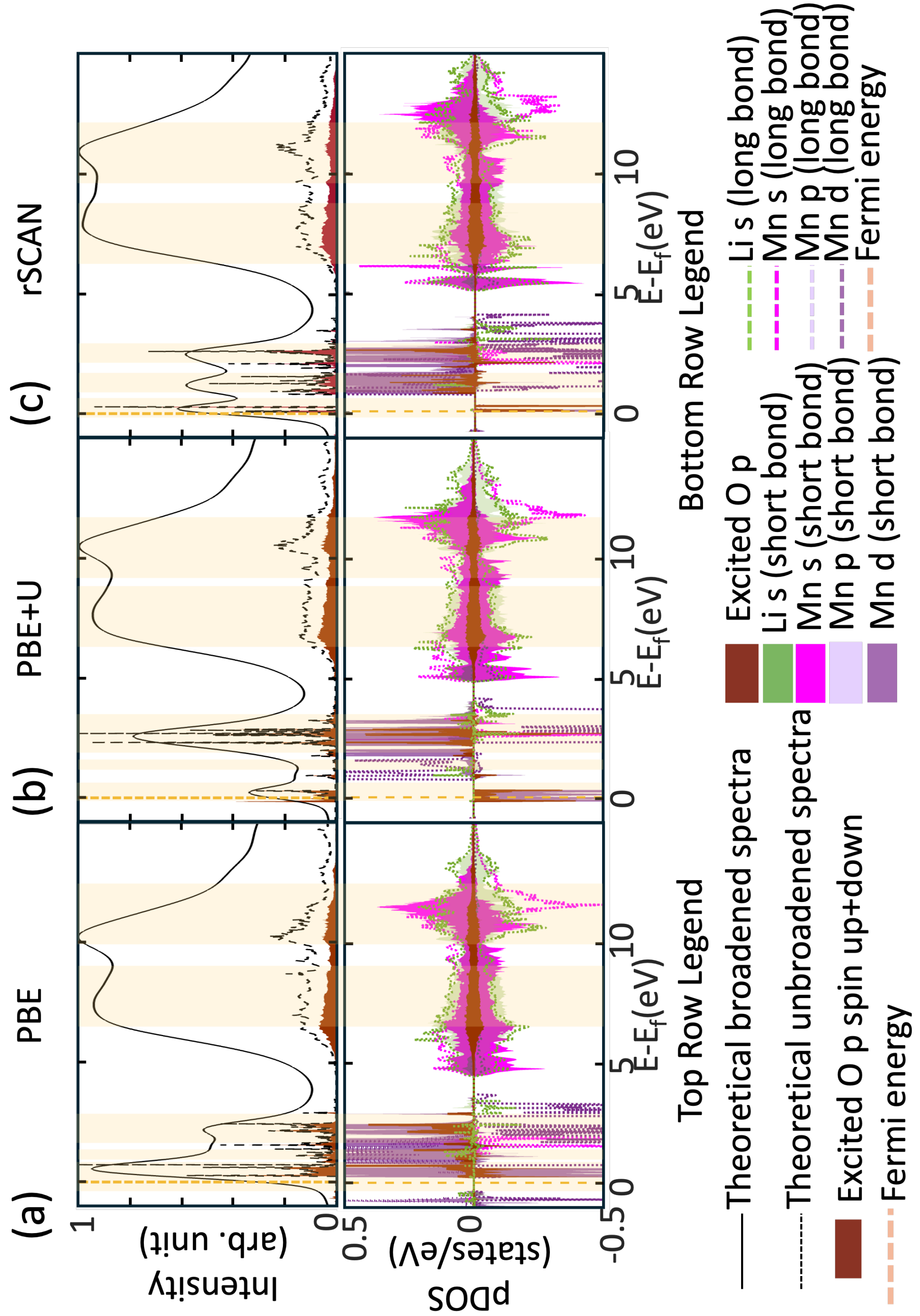

Figure S4: $C2/m$ $LiMnO_2$ AFM pDOS site 1. (a) PBE (b) PBE+U (c) rSCAN. Row 1 is the broadened and unbroadened theoretical spectra compared to the sum of the spin-up and down O-p states of the excited O; Row 2 is the O s and p pDOS of the excited oxygen, along with pDOS of nearest neighbor Li and Mn (short JT bond) and the second nearest neighbor Li and Mn (long JT bond). E_f is the Fermi energy.

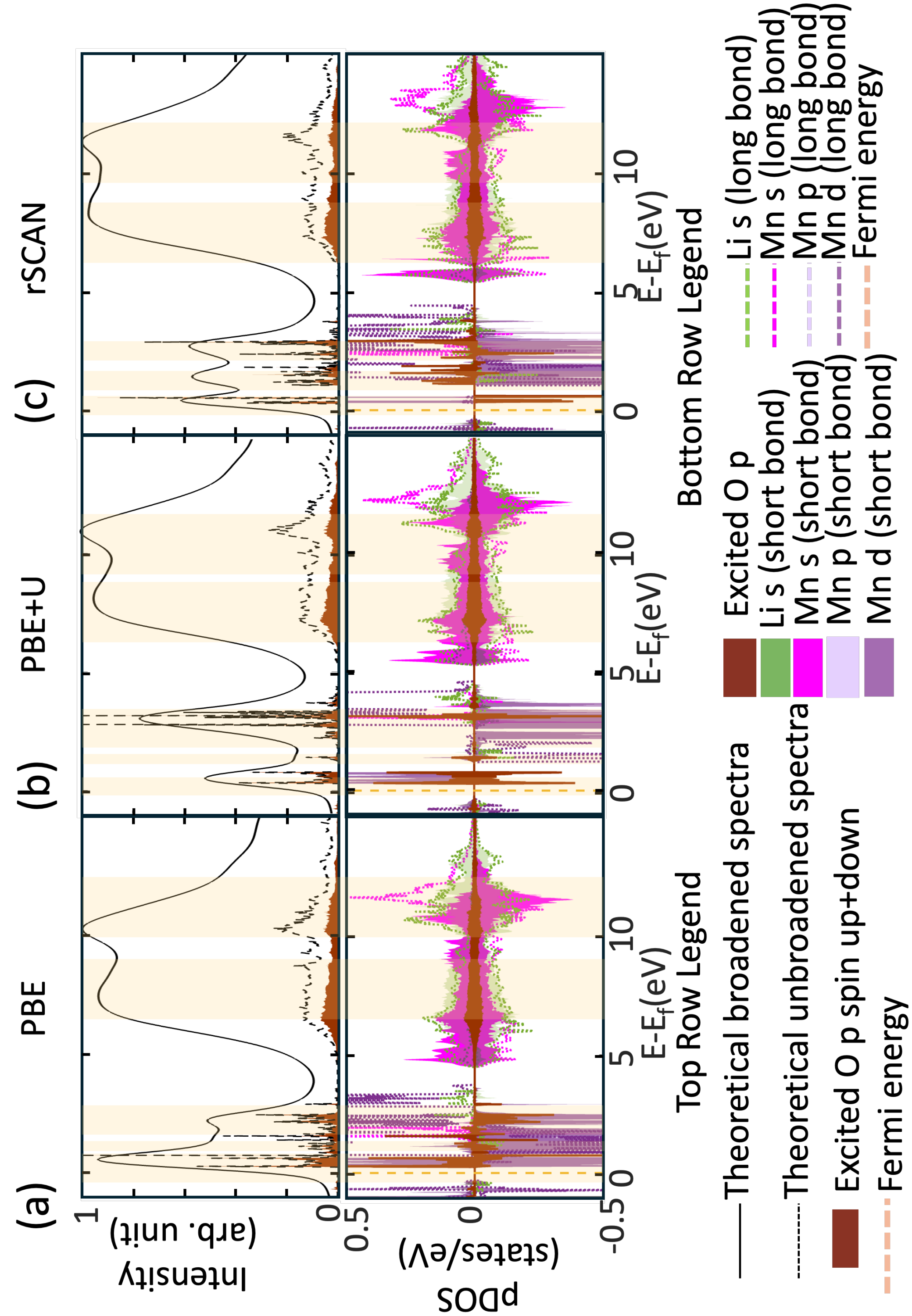

Figure S5: Monoclinic m-LiMnO$_2$ AFM pDOS site 2. (a) PBE (b) PBE+U (c) rSCAN. Row 1 is the broadened and unbroadened theoretical spectra compared to the sum of the spin-up and down O-p states of the excited O; Row 2 is the O s and p pDOS of the excited oxygen, along with pDOS of nearest neighbor Li and Mn (short JT bond) and the second nearest neighbor Li and Mn (long JT bond). E$_f$ is the Fermi energy.

Effect of rSCAN on geometric and electronic structure

The effect of rSCAN stretching out states could be due to rSCAN giving a better structure to start with, or because rSCAN is better at capturing the core-loss spectrum. To disentangle the effect of rSCAN on geometric and electronic structure, we compare the geometry optimised structure parameters given by PBE and rSCAN to the experimental values. We select $LiCoO_2$ and $LiMnO_2$ for this comparison, as the ground state structure of these materials are not as debated as that of $LiNiO_2$. The result of this comparison is shown in Tables S2 and S3. Both comparisons show that rSCAN results in a structure smaller than and also closer to that of experimental values, relative to PBE.

Table S2: Comparisons of theoretical and experimental structure parameters of m-$LiMnO_2$

	a (Å)	b (Å)	c (Å)	$\alpha(^\circ)$	$\beta(^\circ)$	$\gamma(^\circ)$
rSCAN	5.433	2.780	5.355	90.000	116.359	90.000
PBE	5.543	2.810	5.482	90.000	117.265	90.000
Experimental	5.439	2.809	5.388	90.000	116.006	90.000
rSCAN diff	−0.006	−0.029	−0.033	0.000	0.353	0.000
PBE diff	0.104	0.001	0.094	0.000	1.259	0.000

Table S3: Comparisons of theoretical and experimental structure parameters of $LiCoO_2$

	a (Å)	b (Å)	c (Å)	$\alpha(^\circ)$	$\beta(^\circ)$	$\gamma(^\circ)$
rSCAN	2.812	2.812	13.89	90.000	90.000	120.00
PBE	2.851	2.851	14.04	90.000	90.000	120.000
Experimental	2.816	2.816	14.05	90.000	90.000	90.000
rSCAN diff	−0.004	−0.004	−0.169	0.000	0.000	0.000
PBE diff	0.035	0.035	−0.010	0.000	0.000	0.000

To understand the impact of rSCAN on the electronic structure, non core-hole core loss calculations are conducted to see whether rSCAN results in a stretching of states relative to PBE even with the same structure. Figure S6 shows the result of this comparison, taking $LiCoO_2$ as an example, depicting spectra arising from a PBE spectral calculation on a PBE geometry optimised structure (blue), PBE spectral calculation on an rSCAN geometry optimised structure (red dotted), rSCAN spectral calculation on an rSCAN geometry optimised

structure (red). As both the spectra in red show, given the same structure, rSCAN still results in a stretching of states that is seen between the solid blue and red spectra.

Thus, rSCAN results in a stretching of states that gives rise to better description of the spectral positions in the edge region, even with the same structure.

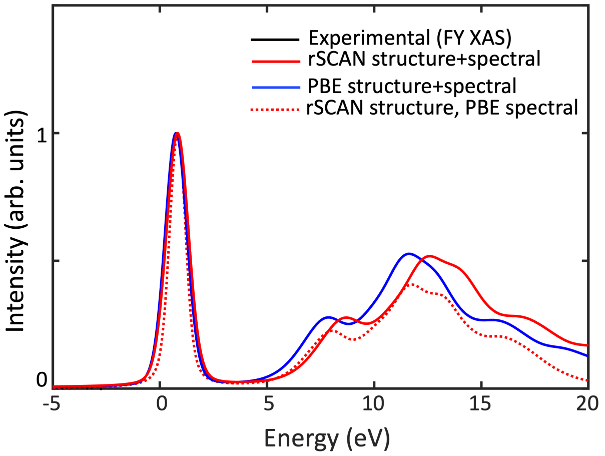

Figure S6: Disentangling the effect that rSCAN has on geometric and electronic structure is depicted. The graph depicts non core-hole core loss calculations arising from $LiCoO_2$.

m-$LiMnO_2$ characterization

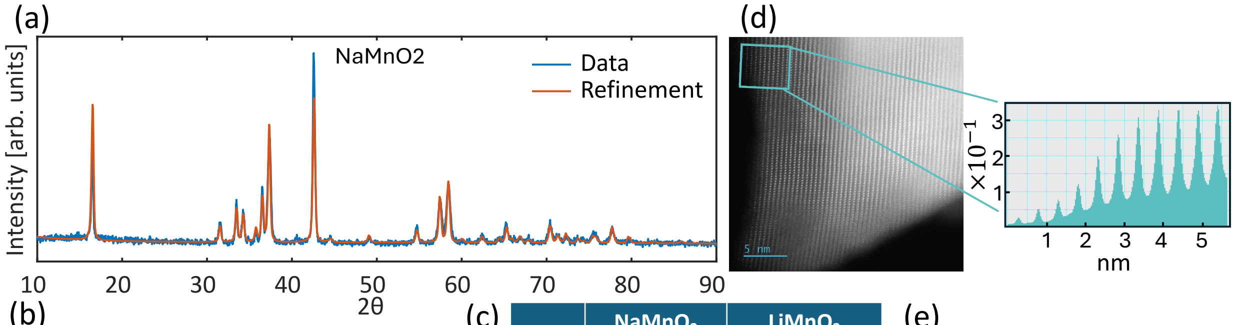

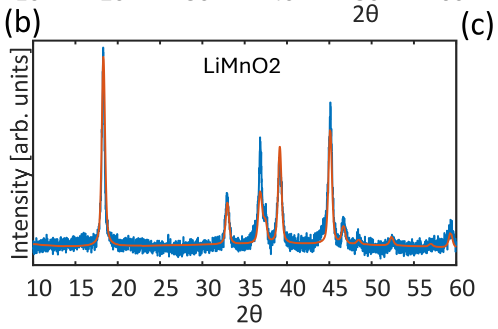

(c)

	$NaMnO_2$	$LiMnO_2$
a	5.690273 Å	5.451417 Å
b	2.854765 Å	2.815771 Å
c	5.819822 Å	5.399298 Å
α	90°	90°
β	113.3925°	116.0856°
γ	90°	90°
χ^2	1.70591	1.08794
R_w	11.7 %	9.8 %

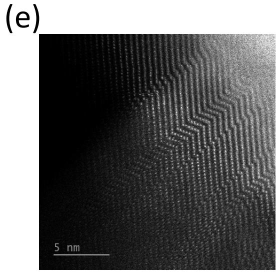

Figure S7: Monoclinic m-$LiMnO_2$ characterization. The XRD and Reitveld refinements are depicted for (a) $NaMnO_2$ and (b) $LiMnO_2$, and the Reitveld refinement fitting parameters are providing in (c). The ADF STEM images depicting $LiMnO_2$ are depicted in (d)-(e).

Powder X-ray Diffraction (XRD) data were collected using a Cu-source Rigaku Miniflex 600 diffractometer housed within an N2-filled glovebox to avoid air exposure of the samples during measurement. The X-rays were un-monochromated but the instrument was fitted with a Ni foil to filter out the K-beta radiation.

The electron microscopy study was carried out on an aberration-corrected JEOL ARM200F electron microscope operated at 200 kV. In ADF STEM imaging, the convergence semi-angle was 22.4 mrad and the inner and outer collection angle was 72.8–271 mrad.
